# Extensive virologic and immunologic characterization in an HIV-infected individual following allogeneic stem cell transplant and analytic cessation of antiretroviral therapy: A case study

**DOI:** 10.1371/journal.pmed.1002461

**Published:** 2017-11-28

**Authors:** Nathan W. Cummins, Stacey Rizza, Mark R. Litzow, Stephane Hua, Guinevere Q. Lee, Kevin Einkauf, Tae-Wook Chun, Frank Rhame, Jason V. Baker, Michael P. Busch, Nicolas Chomont, Patrick G. Dean, Rémi Fromentin, Ashley T. Haase, Dylan Hampton, Sheila M. Keating, Steven M. Lada, Tzong-Hae Lee, Sekar Natesampillai, Douglas D. Richman, Timothy W. Schacker, Stephen Wietgrefe, Xu G. Yu, Joseph D. Yao, John Zeuli, Mathias Lichterfeld, Andrew D. Badley

**Affiliations:** 1 Division of Infectious Diseases, Mayo Clinic, Rochester, Minnesota, United States of America; 2 Division of Hematology, Mayo Clinic, Rochester, Minnesota, United States of America; 3 Ragon Institute of MGH, MIT and Harvard, Cambridge, Massachusetts, United States of America; 4 HIV Immunovirology Unit, Laboratory of Immunoregulation, National Institute of Allergy and Infectious Diseases, National Institutes of Health, Bethesda, Maryland, United States of America; 5 Abbott Northwestern Hospital, Allina Health, Minneapolis, Minnesota, United States of America; 6 Department of Microbiology and Immunology, University of Minnesota, Minneapolis, Minnesota, United States of America; 7 Division of Infectious Diseases, Hennepin County Medical Center, Minneapolis, Minnesota, United States of America; 8 Blood Systems Research Institute, San Francisco, California, United States of America; 9 Department of Laboratory Medicine, University of California, San Francisco, San Francisco, California, United States of America; 10 Centre de Recherche du CHUM, University of Montreal Hospital Centre, Montreal, Canada; 11 Department of Microbiology, Infectious Diseases and Immunology, University of Montreal, Montreal, Canada; 12 Division of Transplantation Surgery, Mayo Clinic, Rochester, Minnesota, United States of America; 13 University of California, San Diego, San Diego, California, United States of America; 14 VA San Diego Healthcare System, San Diego, California, United States of America; 15 Department of Medicine, University of Minnesota, Minneapolis, Minnesota, United States of America; 16 Infectious Disease Division, Brigham and Women’s Hospital, Boston, Massachusetts, United States of America; 17 Department of Laboratory Medicine and Pathology, Mayo Clinic, Rochester, Minnesota, United States of America; University of Melbourne, AUSTRALIA

## Abstract

**Background:**

Notwithstanding 1 documented case of HIV-1 cure following allogeneic stem cell transplantation (allo-SCT), several subsequent cases of allo-SCT in HIV-1 positive individuals have failed to cure HIV-1 infection. The aim of our study was to describe changes in the HIV reservoir in a single chronically HIV-infected patient on suppressive antiretroviral therapy who underwent allo-SCT for treatment of acute lymphoblastic leukemia.

**Methods and findings:**

We prospectively collected peripheral blood mononuclear cells (PBMCs) by leukapheresis from a 55-year-old man with chronic HIV infection before and after allo-SCT to measure the size of the HIV-1 reservoir and characterize viral phylogeny and phenotypic changes in immune cells. At day 784 post-transplant, when HIV-1 was undetectable by multiple measures—including PCR measurements of both total and integrated HIV-1 DNA, replication-competent virus measurement by large cell input quantitative viral outgrowth assay, and in situ hybridization of colon tissue—the patient consented to an analytic treatment interruption (ATI) with frequent clinical monitoring. He remained aviremic off antiretroviral therapy until ATI day 288, when a low-level virus rebound of 60 HIV-1 copies/ml occurred, which increased to 1,640 HIV-1 copies/ml 5 days later, prompting reinitiation of ART. Rebounding plasma HIV-1 sequences were phylogenetically distinct from proviral HIV-1 DNA detected in circulating PBMCs before transplantation. The main limitations of this study are the insensitivity of reservoir measurements, and the fact that it describes a single case.

**Conclusions:**

allo-SCT led to a significant reduction in the size of the HIV-1 reservoir and a >9-month-long ART-free remission from HIV-1 replication. Phylogenetic analyses suggest that the origin of rebound virus was distinct from the viruses identified pre-transplant in the PBMCs.

## Abstract 2

Andrew Badley and colleagues study the viral reservoir in an individual with HIV infection after allogeneic stem cell transplantation.

## Author summary

**Why was this study done?:**

- Currently there is no cure for HIV infection.
- The only previously documented case of HIV cure occurred in the setting of stem cell transplantation for acute myeloid leukemia. However, other similar cases have not resulted in HIV cure.
- This observational study was done to further describe in detail the effects of allogeneic stem cell transplantation on residual HIV in a patient being treated for acute lymphoblastic leukemia.

**What did the researchers do and find?:**

- We prospectively collected blood and tissue samples from before and after stem cell transplantation and measured the HIV reservoir size using multiple complementary techniques.
- We found that the size of the HIV reservoir decreased substantially after transplantation to levels at or below the limit of detection of most assays.
- We observed a prolonged remission from HIV rebound after antiretroviral treatment interruption in the post-transplant period. The genetic sequence of the rebounding virus in the blood clustered closely with sequences from blood prior to treatment interruption.

**What do these findings mean?:**

- These findings affirm that allogeneic stem cell transplantation can profoundly decrease the size of the HIV reservoir.
- However, current technologies for measuring reservoir size in blood are insufficiently sensitive to predict HIV cure.
- Until new biomarkers of HIV cure are developed, the decision to discontinue antiretroviral therapy after allogeneic stem cell transplantation to assess a possible cure should be undertaken cautiously.

## Introduction

Since identification of the human immunodeficiency virus (HIV-1) as the causative agent for acquired immunodeficiency syndrome (AIDS), more than 70 million people have been infected, and an estimated 36 million people live with HIV-1 today [1]. Basic science advances in the understanding of HIV-1 have occurred at an unprecedented pace, allowing the development of numerous antiretroviral (ARV) agents, and advances in clinical science have determined optimal ways of using these drugs to reduce the morbidity and mortality associated with HIV-1 infection. Notwithstanding these impressive successes in the management of HIV-1, individuals who receive effective ARV therapy nonetheless have excess mortality compared to HIV-1 negative populations, due to the effects of inflammation and accelerated aging, manifested as increased risk of cardiovascular, metabolic, and malignant diseases [2]. Thus, a cure for HIV-1 infection is needed [3].

To date, only 1 case, known as the “Berlin patient,” has been cured of HIV-1 [4] by total myeloablative chemotherapy and total body irradiation treatment for acute myeloid leukemia, followed by 2 allogeneic stem cell transplants using cells from a donor who was homozygous for *CCR5* Δ32, rendering the donor cells resistant to R5-tropic HIV-1 infection. Now, more than 10 years after stopping his anti-HIV medications, the Berlin patient remains free from viral rebound, and ultrasensitive assays have repeatedly failed to detect definitive evidence of viral persistence [5].

Unfortunately, other cases of HIV-1-infected patients undergoing allogeneic stem cell transplants (with cells from *CCR5* wild-type donors) have not had durable remissions from HIV-1 rebound following analytic treatment interruption (ATI), with 2 such cases experiencing viral rebound at 12 and 32 weeks post-ATI [6]. Most likely, allogeneic stem cell transplantation (allo-SCT) in these 2 patients significantly decreased, but did not fully eliminate, latently HIV-infected cells, so that viral rebound ignited by persisting viral reservoirs ultimately occurred. However, this interpretation does not exclude the possibility that allogeneic hematopoietic stem cell transplants may, at least in certain cases, induce a more profound or near-complete elimination of viral reservoirs, to enable a long-term drug-free remission of HIV-1 infection.

To explore that possibility, we took advantage of the opportunity to study viral and immune dynamics in an HIV-1 positive patient who, following treatment with prolonged suppressive ARV therapy, developed acute lymphoblastic leukemia and underwent allo-SCT with concurrent ARV therapy. Herein we report a comprehensive analysis of viral and immune parameters occurring after allo-SCT, before and after an ATI.

## Methods

### Study participant

A formal prospective analysis plan was not in place for this study prior to onset. Study visits were determined by routine clinical care, and acquisition of research samples followed previously approved protocols as follows. Following informed consent, and Mayo Clinic Institutional Review Board approval (protocol number 13–005646), the patient underwent leukapheresis on day −11 pre-transplant and days +142, +265, and +888 post-transplant. Leukapheresis was performed on Fenwal Amicus apheresis systems (version 3.1; Fenwal, Lake Zurich, IL, US) using peripheral venous access. ATI was performed under IRB protocol number 15–001678. The patient provided verbal informed consent to reporting and publication of his case history.

Peripheral CD4 T cell counts were measured as previously described [7]. Plasma HIV-1 viral load was measured using the COBAS AmpliPrep/COBAS TaqMan HIV-1 Test, version 2.0 (Roche Molecular Systems, Branchburg, NJ, US). Clinical HIV-1 proviral DNA was measured using the Amplicor HIV-1 DNA Test, version 1.5 (Roche Diagnostics, Indianapolis, IN, US). Presence of HIV-1 antibodies in serum was confirmed using GS HIV-1 Western Blot (Bio-Rad Laboratories, Redmond, WA, US).

### Quantitative viral outgrowth assays

Resting CD4 T cells were isolated by negative selection and verified to be CD4^+^, CCR7^+^, CD27^+^, CD8^−^, CD25^−^, HLA-DR^−^, and CD11b^−^. Quantitative viral outgrowth assays (QVOAs) were then performed as previously described [8]. Cultures were analyzed on day 15 for the presence of p24 in the culture supernatant, and the frequency of infection of resting CD4 T cells was determined using maximum likelihood estimates (expressed as number of infectious units per million resting CD4 T cells, similar to what we and others have previously reported).

### Total HIV DNA and RNA by quantitative PCR

Total HIV-1 DNA in CD4 T cells was measured by real-time PCR as previously described [9]. Cell-associated HIV-1 RNA was measured using the Roche AmpliPrep Kit (detection limit is 20 copies). RNA was isolated using the RNeasy Mini Kit (Qiagen, Venlo, Netherlands) per manufacturer’s protocol.

### Total HIV-1 DNA by droplet digital PCR

Droplet digital PCR (ddPCR) analysis was performed as previously described [10]. Briefly, peripheral blood mononuclear cell (PBMC) DNA was extracted using the Qiagen QIAamp DNA Blood Maxi Kit according to manufacturer’s instructions. Approximately 24,000 ng of total PBMC DNA was assayed by HIV-1 *gag* [11] and HIV-1 *pol* [12] duplex ddPCR using previously published TaqMan assays and Bio-Rad QX200 reagents. RPP30 DNA cell normalizer was measured in a separate ddPCR reaction. ddPCR droplet data were acquired and analyzed by Bio-Rad QuantaSoft software and are expressed as HIV copies per 1 million cells; limits of detection and 95% confidence intervals were calculated based on Poisson statistics and total number of droplet events analyzed. In total, 24 HIV-1 DNA ddPCR replicates, with approximately 1,000 ng per ddPCR replicate, and >250,000 droplet events were analyzed.

### Integrated HIV-1 DNA in CD4 T cell subsets

CD4 T cell subsets (e.g., TN, TCM, TTM, and TEM) were sorted based on the expression of CD45RA, CCR7, and CD27, as described previously [13]. Sorted cells were subjected to proteinase K digestion, and the frequency of cells harboring integrated HIV-1 DNA was determined as previously described [14].

### In situ hybridization

After deparaffinization with xylene, and rehydration through graded ethanols, tissue sections were treated with HCl, triethanolamine, digitonin, and 4 mcg/ml proteinase K, as previously described [15]. After acetylation with acetic anhydride and dehydration, tissue sections were hybridized at 45°C overnight with a ^35^S-labeled riboprobe and 0.5 mM aurintricarboxylic acid in the hybridization mix. After extensive washes and ribonuclease treatment, tissue sections were dehydrated, coated in Kodak NTB emulsion diluted with 10% glycerol and 0.1 M ammonium acetate, exposed at 4°C for 7–14 days, and developed and fixed as previously described [15].

### Microchimerism

Highly sensitive allele-specific PCR assays targeting HLA and insertion–deletion polymorphisms unique to the patient or donor were used to determine levels of host microchimerism in blood (the proportion of residual host PBMCs after hematopoietic stem cell transplantation), as previously described [16,17]. The microchimerism assay is highly specific and sensitive to a single copy of target DNA, allowing detection of host cells present as a very low proportion of the PBMC population, depending on the number of cells surveyed [17].

### Immunophenotyping by flow cytometry

PBMCs were stained with selected monoclonal antibodies labeled with defined combinations of fluorescent dyes. Cells were then washed, fixed, washed again, and analyzed on a Fortessa flow cytometer, using standard protocols. Data were analyzed using FlowJo software (Treestar, Ashland, OR, US).

### HIV antibody measurement

The gp41-detecting Limiting Antigen (LAg)–Avidity EIA (Sedia Biosciences, Portland, OR, US) was performed as previously described [18,19]. In brief, assay controls and HIV-positive specimens were diluted 1:101 in specimen diluent, and 100 μl of calibrator, controls, or specimens was added to antigen-coated plates and incubated. Plates were washed 4 times with 1× wash buffer to remove unbound antibodies. A pH 3.0 buffer was added to each well to dissociate low-avidity antibodies. Plates were developed, and the optical density (OD) was read using a spectrophotometer (microplate reader; Molecular Devices, Sunnyvale, CA, US). Raw OD for each specimen was normalized using the calibrator OD on each plate as a ratio, such that normalized OD = OD of specimen/median OD of calibrator.

### Viral sequencing

Genomic DNA was extracted from indicated cell populations using the Qiagen DNeasy Blood & Tissue Kit and diluted to single-genome levels based on Poisson distribution statistics of HIV-1 *gag* amplification results. Subsequently, single-genome viral gene amplification was performed using Invitrogen Platinum Taq (Invitrogen, Carlsbad, CA, US) and nested primers spanning near full-length HIV-1 (HXB2 positions 638–9632). Primers were previously published [20] except for a modified nested forward primer: 5′-GCGCCCGAACAGGGACYTGAAARCGAAAG-3′. PCR products were visualized by agarose gel electrophoresis and subjected to Illumina MiSeq sequencing. Resulting short reads were de novo assembled and aligned to HXB2. Integrity of full-length sequences was determined using an automated in-house pipeline written in R scripting language [21]. Presence/absence of APOBEC-3G/3F-associated hypermutations was determined using Los Alamos HIV Sequence Database Hypermut 2.0 [22]. Multiple sequence alignments were performed using MUSCLE [23]. Genetic distances between sequences were examined using Clustal X–generated neighbor joining algorithms [24]. For the analysis of plasma HIV-1 sequences, plasma HIV-1 RNA was transcribed to cDNA using standard procedures, diluted to single genomes, and subjected to nested PCR with primers annealing to *env* (first round primers: 5′-CACCGGCTTAGGCATCTCCTATGGCAGGAAGAA-3′ and 5′- CATTGGTCTTAAAGGTACCTGAGG-3′; second round primers: 5′-AGAAAGAGCAGAAGACAGTGGCAATGA-3′ and 5′-TTTTGACCACTTGCCACCCAT-3′) and *pol* (first round primers: 5′-TGTACTGAGAGACAGGCTAATTTTT-3′ and 5′-AAACTCCCACTCAGGAATCCAGGT-3′; second round primers: 5′-AGACAGGCTAATTTTTTAGGGAAGAT-3′ and 5′-CACTCAGGAATCCAGGTGGCTT-3′). Subsequently, PCR products were processed by Sanger sequencing; sequence alignments were performed using MUSCLE.

## Results

In June 2013, a 55-year-old HIV-1 positive man was referred to Mayo Clinic for evaluation of B-lineage acute lymphoblastic leukemia. Pre-transplant HIV-1 history is described in Table 1. Briefly, he had been first diagnosed with HIV-1 infection in 1990 and believed his infection occurred in 1982. At the time of diagnosis, his CD4 count was >500 cells/μl (reference range 365–1,437), his plasma HIV-1 RNA viral load was approximately 400 copies/ml, and he did not receive ARV therapy. In 1999, when his CD4 count had declined to approximately 300 cells/μl and his HIV-1 viral load had increased to 10,000 copies/ml, he was started on ritonavir-boosted indinavir and zidovudine/lamivudine. In 2004, he took a drug holiday. When his HIV-1 viral load had increased to approximately 10,000 copies/ml in 2009, he initiated ritonavir-boosted atazanavir and tenofovir/emtricitabine. His regimen was changed to raltegravir and tenofovir/emtricitabine in April 2013 to avoid potential drug–drug interactions with anticipated chemotherapy (as noted below). He tolerated these medications with excellent adherence, and at the time of presentation for leukemia evaluation, his anti-HIV-1 Western blot was positive, with a plasma HIV-1 viral load of 107 copies/ml and a CD4 count of 293 cells/μl (37% of CD3^+^ cells).

**Table 1:** Pre-transplant HIV laboratory test results and antiretroviral treatment history.

Time point	CD4 T cell count (cells/μl)	HIV-1 RNA (copies/ml)	HIV therapy
1990 (HIV diagnosis)	>500	400	None
1999	300	10,000	AZT/3TC, IDV/rtv
2004	>500	Undetectable	Therapy stopped
2009	>500	10,000	TDF/FTC ATV/rtv
6/2013	293	107	TDF/FTC, raltegravir
9/2013 (began chemotherapy for leukemia)	183	25	TDF/FTC, raltegravir, etravirine
10/2013 (allogeneic stem cell transplant on Oct 6)		Detected	TDF/FTC, raltegravir, etravirine

3TC, lamivudine; ATV, atazanavir; AZT, zidovudine; FTC, emtricitabine; IDV, indinavir; rtv, ritonavir; TDF, tenofovir.

In March 2013, he experienced the insidious onset of progressive light-headedness and fatigue, associated with a white blood cell count of 3,400 cells/mm^3^ (reference range, 4,500–11,000) with 52% circulating blasts, hemoglobin 80 g/l (reference range, 135–175), and platelets of 47,000 cells/mm^3^ (reference range, 140,000–440,000). A bone marrow biopsy was 90% cellular with 96% blasts. Flow cytometry showed multiple B-lineage markers including CD20, CD79a, and intranuclear terminal deoxynucleotidyl transferase. Cytogenetics were normal, and fluorescence in situ hybridizations for BCR-ABL and MLL rearrangement were both negative. Cerebrospinal fluid examination showed leukemic blasts, and a CT scan showed mild mediastinal adenopathy up to 14 mm in short-axis dimension, while the spleen was enlarged, at craniocaudal height of 17 cm. The patient received treatment with rituximab and hyper-CVAD (cyclophosphamide, vincristine, doxorubicin, dexamethasone) alternating with high-dose methotrexate and cytarabine beginning in April 2013. Repeat bone marrow biopsy in May 2013 showed 4% circulating blasts and 9% bone marrow blasts, prompting a third cycle of rituximab and hyper-CVAD; a repeat bone marrow biopsy in July was normocellular, with a cellularity of 40%. No morphologic features of acute leukemia were noted. In August 2013, he underwent a fourth cycle of hyper-CVAD with high-dose methotrexate and cytarabine.

In an effort to attain full HIV viral suppression, the patient’s ART regimen was intensified due to persistent low-level viremia (plasma HIV-1 viral load ranging from 90 to 107 copies/ml). In anticipation of myeloablative chemotherapy, his ARV regimen was modified to include etravirine 200 mg twice daily in addition to his current ART regimen of raltegravir and co-formulated tenofovir/emtricitabine. Due to a history of prior gastric bypass, concern for poor drug absorption in the setting of his low-level viremia, and reported decreased raltegravir exposure while on etravirine [25], the raltegravir dose was empirically increased to 600 mg twice daily, where satisfactory 3-hour peak levels were demonstrated (1.11 μg/ml; reference range, 0.67–3.54 μg/ml).

In October 2013, the patient underwent a fludarabine/melphalan reduced-intensity conditioning treatment prior to an HLA-matched, ABO-matched allogeneic peripheral blood stem cell transplant with infusion of 4.47 × 10^6^ CD34^+^ cells/kg from a *CCR5* wild-type donor. Donor and recipient characteristics are listed in Table 2. The patient was placed on tacrolimus and full-dose methotrexate for graft-versus-host disease (GVHD) prophylaxis, and acyclovir, atovaquone, and anidulafungin antimicrobial prophylaxis, with plans to restart trimethoprim/sulfamethoxazole prophylaxis after engraftment, and voriconazole after liver function tests normalized. The patient remained on stable uninterrupted ARVs after peripheral blood stem cell transplantation (PBSCT).

**Table 2:** Donor and recipient characteristics prior to allogeneic stem cell transplantation.

Characteristic	Donor	Recipient
HLA type	A*03,24	A*03,24
	B*07,27	B*07,27
	Cw*02,07	Cw*02,07
	DRB1*04,04	DRB1*04,04
	DRw*53,53	DRw*53,53
	DRB4*01,01	DRB4*01,01
	DQ*07,08	DQ*07,08
	DQB1*03,03	DQB1*03,03
*CCR5* genotype	Wild-type	Wild-type
Cytomegalovirus (IgG)	Positive	Positive
Epstein–Barr virus (IgG)	NA	Positive
Toxoplasma (IgG)	NA	Positive

NA, not available.

In January 2014, the patient discontinued his GVHD prophylaxis, and in February 2014, he developed progressive diarrhea, which prompted a diagnostic colonoscopy (day +133 post-transplant); biopsy and pathology revealed mildly increased crypt cell apoptosis in the colon and ileum (consistent with GVHD), and in situ stains for Epstein–Barr virus, adenovirus, and cytomegalovirus were negative. The patient was treated with loperamide for symptomatic management. In March 2014, trimethoprim/sulfamethoxazole was stopped because of low platelets, and in May 2014, the patient was admitted with fever and shortness of breath, and was diagnosed with *Pneumocystis jirovecii* pneumonia, which was treated with high dose trimethoprim/sulfamethoxazole.

**Fig 1:**
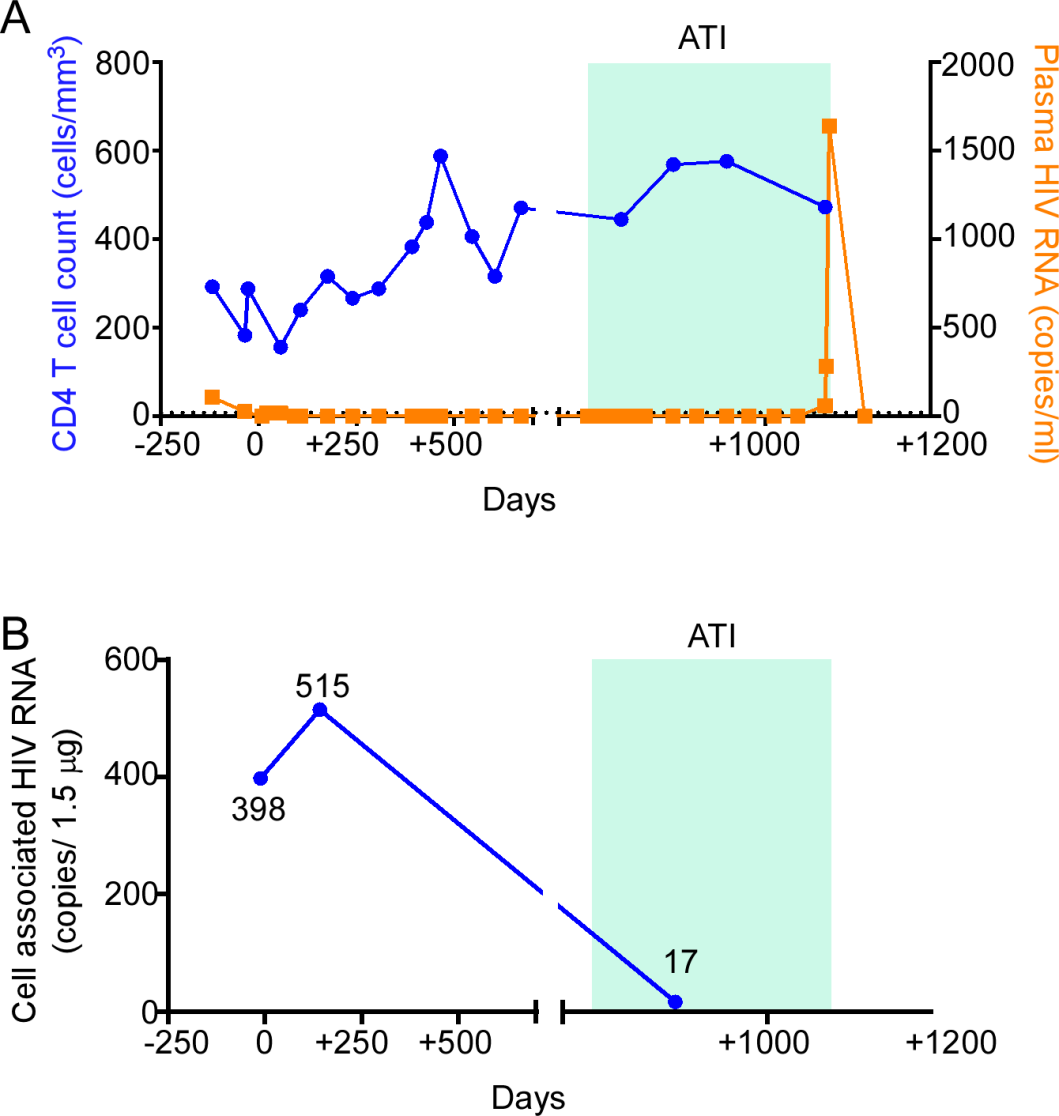
HIV-1 RNA monitoring in the peri-transplant period. (A) CD4 T cell count and plasma HIV-1 RNA were measured in the pre- and post-transplant period. (B) Cell-associated HIV-1 RNA was measured in isolated CD4 T cells from leukapheresis samples on the days indicated. ATI, analytic treatment interruption.

An ATI was started according to an IRB-approved protocol on day +784 post-transplantation (1 December 2015) (Fig 1A). HIV-1 remained persistently undetectable by multiple measures (see below). Plasma HIV-1 RNA was monitored every 2 weeks for 12 weeks, then every 4 weeks thereafter, and remained undetectable (limit of detection at 20 copies/ml, COBAS AmpliPrep/COBAS TaqMan HIV-1 Test, version 2.0). However, at day 288 of the ATI, the patient experienced asymptomatic viral rebound, with a plasma HIV-1 RNA viral load of 60 copies/ml. The plasma HIV-1 RNA viral load rose to 283 copies/ml on ATI day 289 and to 1,640 copies/ml on ATI day 293, prompting reinstitution of ARV therapy, according to the clinical ATI protocol. Resistance testing by viral genotype revealed no mutations associated with ARV drug resistance. The patient denied risk factors for new HIV exposures. Reinstitution of ARV therapy resulted in suppression of detectable viral replication after 4 weeks, and the patient’s hematologic malignancy remains in full remission at the time of this publication.

### HIV-1 RNA monitoring

HIV-1 RNA was detectable in plasma at days −119, −35, +20, and +56 of the peri-transplant period (107, 25, <20, and <20 copies/ml, respectively; lower limit of detection 10 copies/ml and lower limit of quantification 20 copies/ml); beginning 91 days after transplant (day +91), plasma RNA remained undetectable until day +1,072, which was 288 days following initiation of the ATI (Fig 1A). Cell-associated HIV-1 RNA was measured in isolated CD4 T cells sampled pre- and post-transplant (Fig 1B). Cell-associated HIV-1 RNA increased at day +142 (515 copies/1.5 μg RNA) compared to day −11 (397.5 copies/1.5 μg RNA), but was reduced at day +888 (17 copies/1.5 μg RNA) during the ATI.

### HIV-1 DNA monitoring

We next estimated the size of the HIV-1 reservoir over time by measuring HIV-1 DNA in CD4 T cells at multiple time points before and after transplantation using multiple methods. The patient underwent leukapheresis on day −11 pre-transplant and on days +142, +265, +436, and +888 post-transplant to provide cells for analysis.

**Fig 2:**
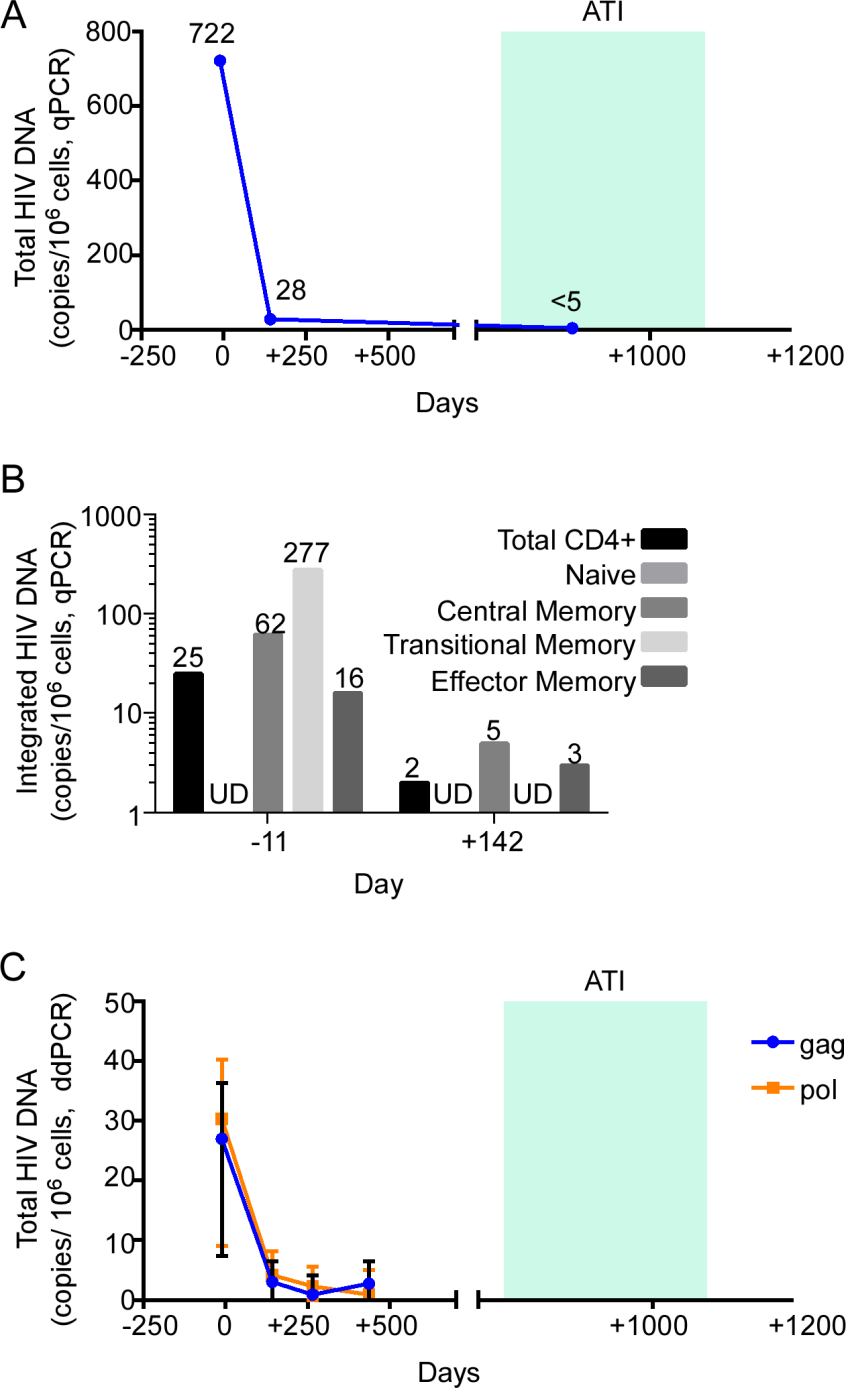
HIV-1 DNA monitoring in the peri-transplant period. (A) Total HIV-1 DNA was measured by quantitative PCR (qPCR) in isolated CD4 T cells from the days indicated. (B) Integrated HIV-1 DNA was measured in sorted bulk CD4 T cells and CD4 T cell subsets from the days indicated. (C) Total HIV-1 DNA was measured by digital droplet PCR (ddPCR) in isolated CD4 T cells from the days indicated. Indicated are point estimates (bars show 95% confidence intervals of the estimates), including for results that were below the lower limit of detection (undetectable [UD]). ATI, analytic treatment interruption.

Total HIV-1 DNA in CD4 T cells was measured by quantitative real-time PCR pre-transplant and on days +142 and +888 post-transplant (Fig 2A). Total HIV-1 DNA decreased from 722 copies/million CD4 T cells pre-transplant to 28 copies/million CD4 T cells at day +142 post-transplant, representing a 96% reduction in HIV-1 DNA. We questioned whether this reduction in HIV-1 DNA in PBMCs was merely secondary to dilution by virtue of replacing HIV-1-DNA-containing recipient cells with uninfected donor cells. Microchimerism evaluation revealed that approximately 8% of DNA in circulating CD4 T cells on day +142 was of recipient origin, indicating that HIV-1 total DNA may have decreased further than expected by hemodilution alone, possibly by preferential loss of HIV-1-DNA-containing cells. Microchimerism analysis of cells from day +265 revealed that 0.0013% of CD4 T cell DNA was of recipient origin. By day +888, total HIV-1 DNA was below the limit of detection (<5 copies/million cells).

We further analyzed which CD4 T cell subsets contained residual HIV. In these studies we measured integrated HIV-1 DNA in sorted memory CD4 T cell subsets from day −11 and day +142 (Fig 2B). We observed a reduction in the frequency of cells harboring integrated HIV-1 DNA between pre-transplant and day +142 post-transplant in the 3 memory CD4 T cell subsets (central memory, transitional memory, and effector memory), with the greatest reduction occurring within the transitional memory CD4 T cell subset (from 277 copies/10^6^ cells to undetectable).

Although most proviral DNA is not replication competent, measurement of HIV-1 DNA permits the largest dynamic range to quantify reduction in HIV-1 reservoir size [26]. We regularly measured HIV-1 DNA over time, using ddPCR and 2 different target primer pairs (Fig 2C). By day +265 post-transplant, HIV-1 DNA measured using *gag* primers decreased to undetectable, while HIV-1 DNA measured using *pol* primers remained detectable at low levels (30.3 copies/10^6^ cells pre-transplant to 2.3 copies/10^6^ cells at day +265 post-transplant). By day +436, HIV-1 *pol* amplification of HIV-1 DNA was undetectable, whereas HIV-1 *gag* amplification of HIV-1 DNA was barely detectable, at 2.7 copies/10^6^ cells.

### HIV-1 quantitative viral outgrowth assay monitoring

**Fig 3:**
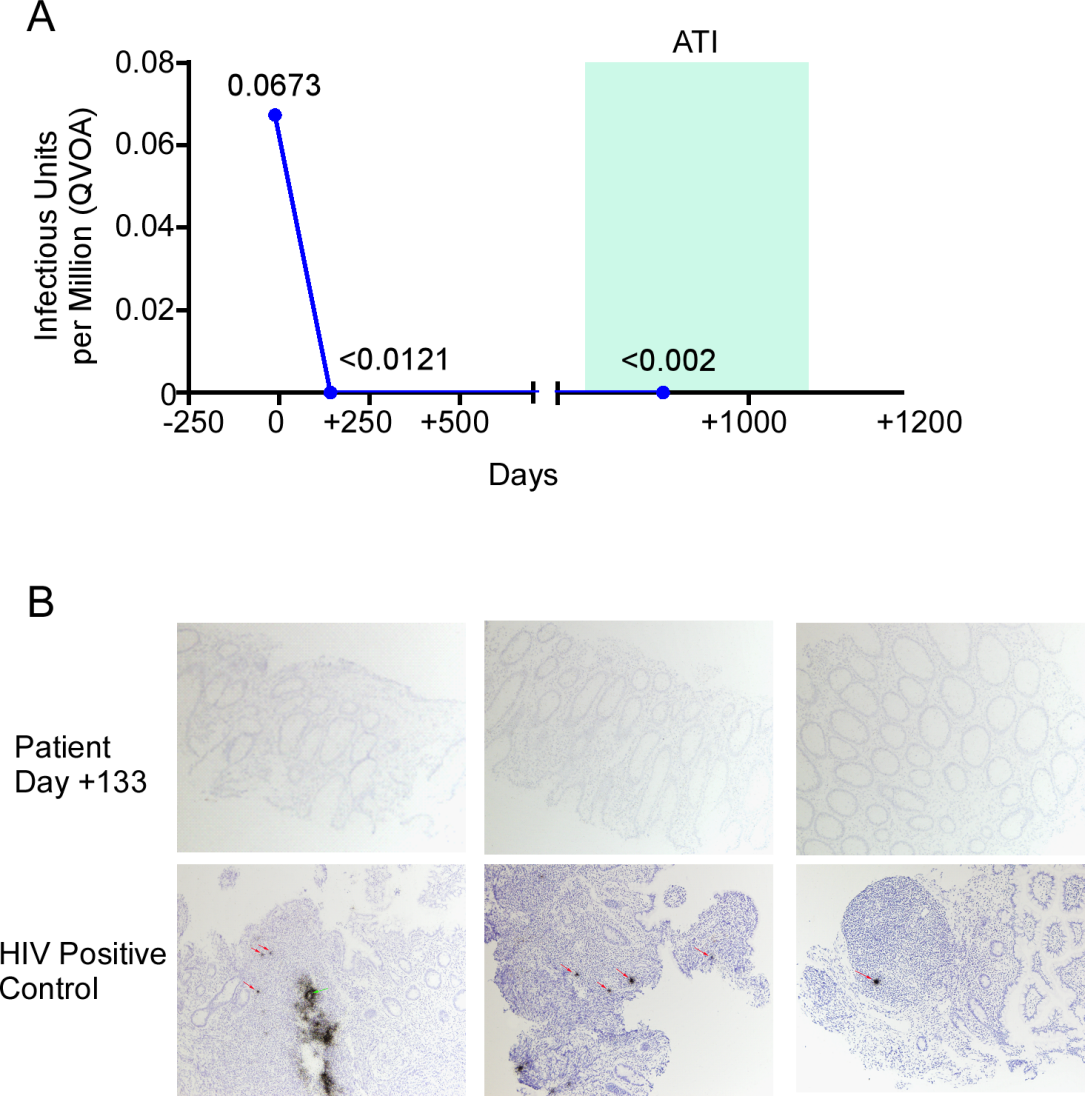
HIV-1 reservoir measurement in the peri-transplant period. (A) Replication-competent virus in isolated peripheral resting CD4 T cells was estimated by quantitative viral outgrowth assay (QVOA) on the days indicated. (B) In situ hybridization for HIV-1 DNA in colon tissue samples obtained on day +133 after transplantation. ATI, analytic treatment interruption.

Of the multiple proposed ways to measure the HIV-1 reservoir size, nucleic amplification approaches have been criticized because they measure both replication-competent viruses as well as replication-incompetent defective viruses [27], which may represent the majority of the measured viruses [28]. Thus, we opted to measure replication-competent HIV-1 by QVOA on CD4 T cells [29]. For this assay, 35 × 10^6^ CD4 T cells from day −11 pre-transplant were cultured at 5 × 10^6^ cells per well and stimulated with αCD3/CD28 antibodies to reactivate any virus present, yielding 2 of 7 wells with detectable p24 antigen production, for an estimated 0.0673 infectious units per million cells (IUPM) (Fig 3A). A similar viral outgrowth assay using 85 × 10^6^ CD4 T cells from day +142 post-transplant yielded 0 of 17 wells producing p24 antigen, for an estimated IUPM of <0.0121. Another viral outgrowth assay performed on cells from day +888 using >400 × 10^6^ cells (while the patient was aviremic during ATI) yielded 0 positive wells, for an estimated IUPM of <0.00235, or less than 1 cell carrying replication-competent HIV-1 per 425 million CD4 T cells.

### HIV-1 In situ hybridization

We used in situ hybridization to asses HIV reservoir size in tissue sections from the diagnostic colon biopsy on day +133 that revealed GVHD. There were no HIV positive cells, and no follicular dendritic cell signal, in a total of 105 biopsy sections (Fig 3B).

### HIV-1 sequencing

**Fig 4:**
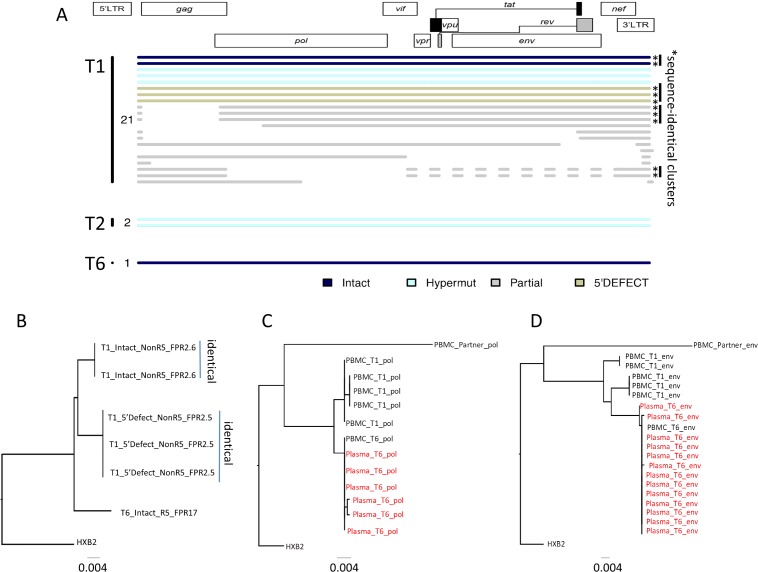
Single-genome, near full-length HIV-1 sequencing in the patient. (A) Diagram summarizing all HIV-1 DNA sequences retrieved from peripheral blood mononuclear cells (PBMCs) at indicated time points (T1: day −11; T2: day +144; T6: day +1078). Asterisks indicate clusters of completely identical proviral sequences. Color coding reflects presence of intact or defective sequences. (B) Phylogenetic tree including all near full-length sequences from indicated time points. Viral tropism (R5 versus nonR5) and geno2pheno false positive rate (FPR) percentage are included for each sequence. (C and D) Phylogenetic trees for viral *env* (HXB2 positions 6271–6889) and *pol* (HXB2 positions 2131–2780) sequences amplified from indicated plasma or PBMC samples collected at T1 or T6. A proviral sequence from the patient’s HIV-1-infected, ART-treated partner is also included. Plasma sequences are denoted in red text.

To more closely examine changes in residual viral reservoirs over time, we conducted single-genome sequencing assays of near full-length proviral HIV-1 DNA, using a recently described experimental approach [30]. Immediately prior to transplantation, we obtained a total of 23 proviral DNA sequences, of which 2 were sequence-intact, corresponding to a frequency of 0.4 intact, near full-length sequences per million PBMCs (Fig 4A). This is lower than previously reported in HIV-1 positive individuals undergoing suppressive ART during chronic infection [30]; however, the patient had previously received chemotherapy for his malignancy, which may account for this low number. Notably, these 2 intact viral sequences were identical, and likely derived from a single HIV-1-infected cell clone. Identical proviral sequences were also observed within the pool of defective viral DNA products, consistent with clonal expansion [30–32] of cells harboring replication-deficient proviral HIV-1 DNA (Fig 4A).

On day +142 after transplantation, we detected 2 hypermutated viral sequences after sampling 577,017 PBMCs, while no viral sequences were detected on days +265, +436, and +888 after transplantation, after analyzing a total of 22,250, 648,217, and 704,583 PBMCs, respectively. At day +1,087 after transplant, when plasma viral rebound was noted, we detected a single near full-length, intact proviral sequence in a total of 3,737,467 analyzed PBMCs, corresponding to an extremely low viral reservoir size in peripheral blood. No defective viral sequences were observed at this time. Interestingly, this viral sequence showed remarkable phylogenetic distance to proviral sequences isolated prior to transplantation and had predicted R5 tropism, while pre-transplant intact sequences were non-R5 tropic (Fig 4B). Correspondingly, we noted that *env* and *pol* sequences from rebounding plasma virus were phylogenetically closely related to the contemporaneous proviral DNA sequence, but exhibited considerable phylogenetic distance to proviral HIV-1 DNA detected prior to transplantation (Fig 4C and 4D). A sequence of the patient’s HIV-1-infected ART-treated partner was phylogenetically clearly unrelated to rebounding HIV-1 plasma sequences, making sexually transmitted superinfection an extremely unlikely explanation of the patient’s viral relapse (Fig 4C and 4D). Together, these data suggest that the rebound viremia originated from a viral variant that was not detected in the peripheral blood compartment at any earlier time point, possibly implicating reactivation of an archived provirus harbored by one or more cellular or anatomical reservoirs that were distinct from CD4 T cells circulating prior to transplantation.

Major drug resistance mutations defined by the Stanford HIV Drug Resistance Database (https://hivdb.stanford.edu) algorithm were not detected in any of the HIV sequences from the patient (inclusive of all time points and all of PBMC- and plasma-derived HIV RNA and DNA). Cytotoxic T lymphocyte (CTL) escape mutation analyses were not performed, given that HIV-1-specific CTLs were only very weakly detected in the patient (see below).

### Cellular immune responses

**Fig 5:**
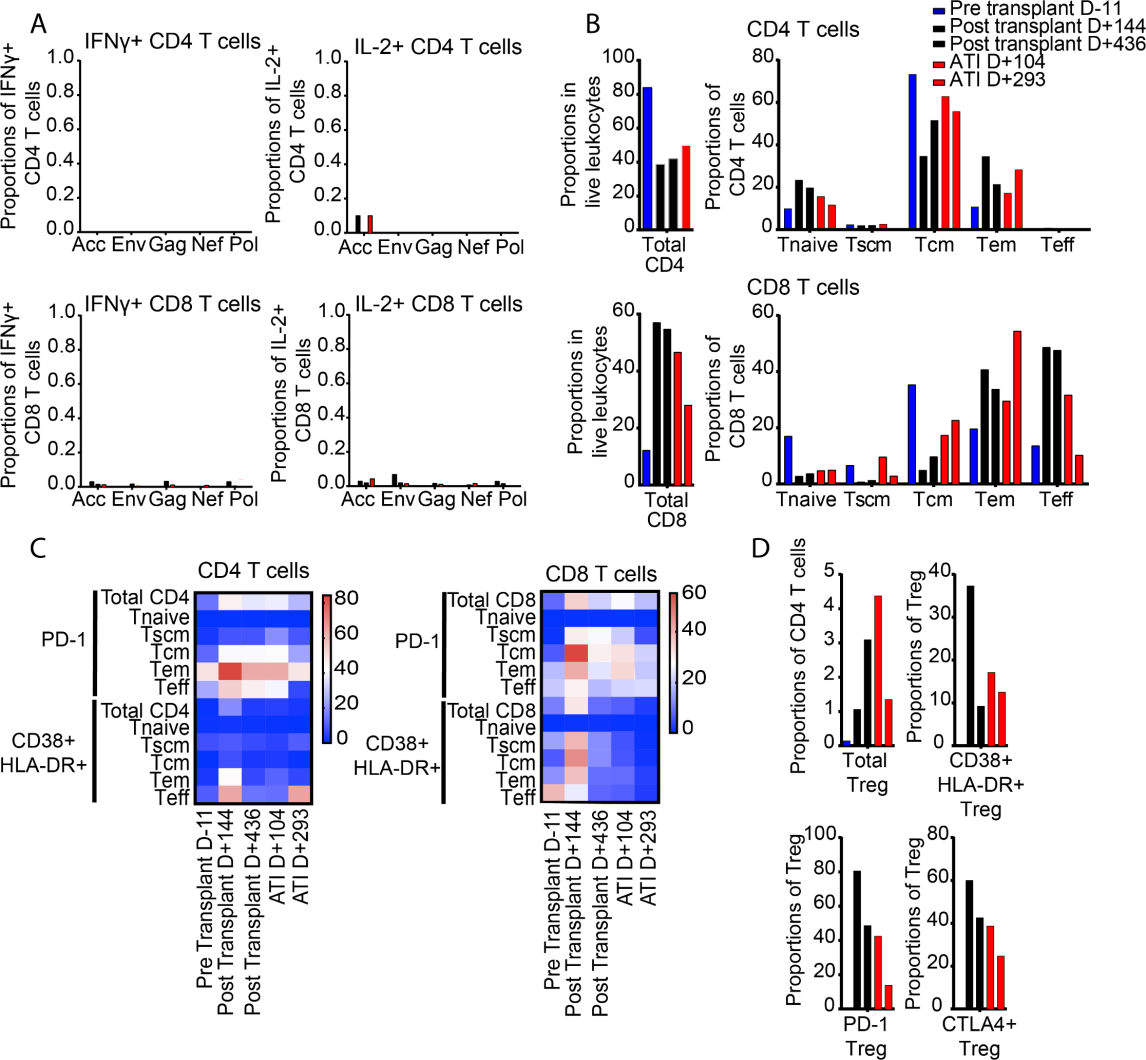
Dynamics of CD4 and CD8 T cell responses in the described patient. (A) Proportions of CD4 (upper plots) and CD8 (lower plots) T cells specific for the indicated HIV-1 gene product. Color coding reflects time of sample collection. HIV-1-specific T cell responses were identified based on antigen-specific IFNγ secretion (left plots) or IL-2 secretion (right plots). (B) Proportions of total CD4 and CD8 T cells, and indicated T cell subsets, within CD4 and CD8 T cells. (C) Heatmaps reflecting the longitudinal evolution of the proportions of indicated CD4 (left plot) and CD8 (right plot) T cell subsets expressing PD-1 or CD38/HLA-DR. (D) Proportions of FoxP3+ regulatory T cells with indicated phenotypic characteristics. ATI, analytic treatment interruption; Tcm, central memory T cells; Teff, terminally differentiated T cells; Tem, effector memory T cells; Treg, regulatory T cells; Tscm, T memory stem cells.

HIV-1-specific CD8 T cell responses can effectively restrict HIV-1 replication by MHC class I–restricted cytolysis and represent an important correlate of antiviral immune protection in individuals with natural control of HIV-1, specifically when restricted by HLA-B27 [33,34], an MHC class I allele present in the recipient and the donor of the hematopoietic stem cells in this case. To analyze HIV-1-specific T cell responses in our patient, we stimulated PBMCs with pools of peptides corresponding to individual HIV-1 gene products, followed by quantification of antigen-induced intracellular cytokine production. These results showed barely detectable HIV-1-specific CD8 T cell responses at all analyzed time points (Fig 5A). A similar observation was made for HIV-1-specific CD4 T cell responses, most of which also remained under the threshold of detection by flow cytometry (Fig 5A). The total number of CD4 cells was significantly expanded prior to transplantation at the expense of CD8 T cells, but CD4:CD8 T cell ratios improved during the subsequent disease process, towards an age-appropriate naïve and memory cell distribution [35] in both the CD4 and CD8 T cells (Fig 5B). Notably, expression of cellular activation markers and immune checkpoints on total CD4 and CD8 T cells was strongly upregulated 144 days after transplantation, the time point associated with his diagnosis of GVHD (Fig 5C). Corresponding to these findings, we observed that CD25+ CD127− FoxP3+ regulatory CD4 T cells were infrequently detected prior to transplantation, followed by a rapid increase of regulatory T cell (Treg) frequencies during the post-transplantation period; expression of immune checkpoint and activation markers on Tregs was most obvious at the time of clinical GVHD (Fig 5D).

### Innate immune cells

**Fig 6:**
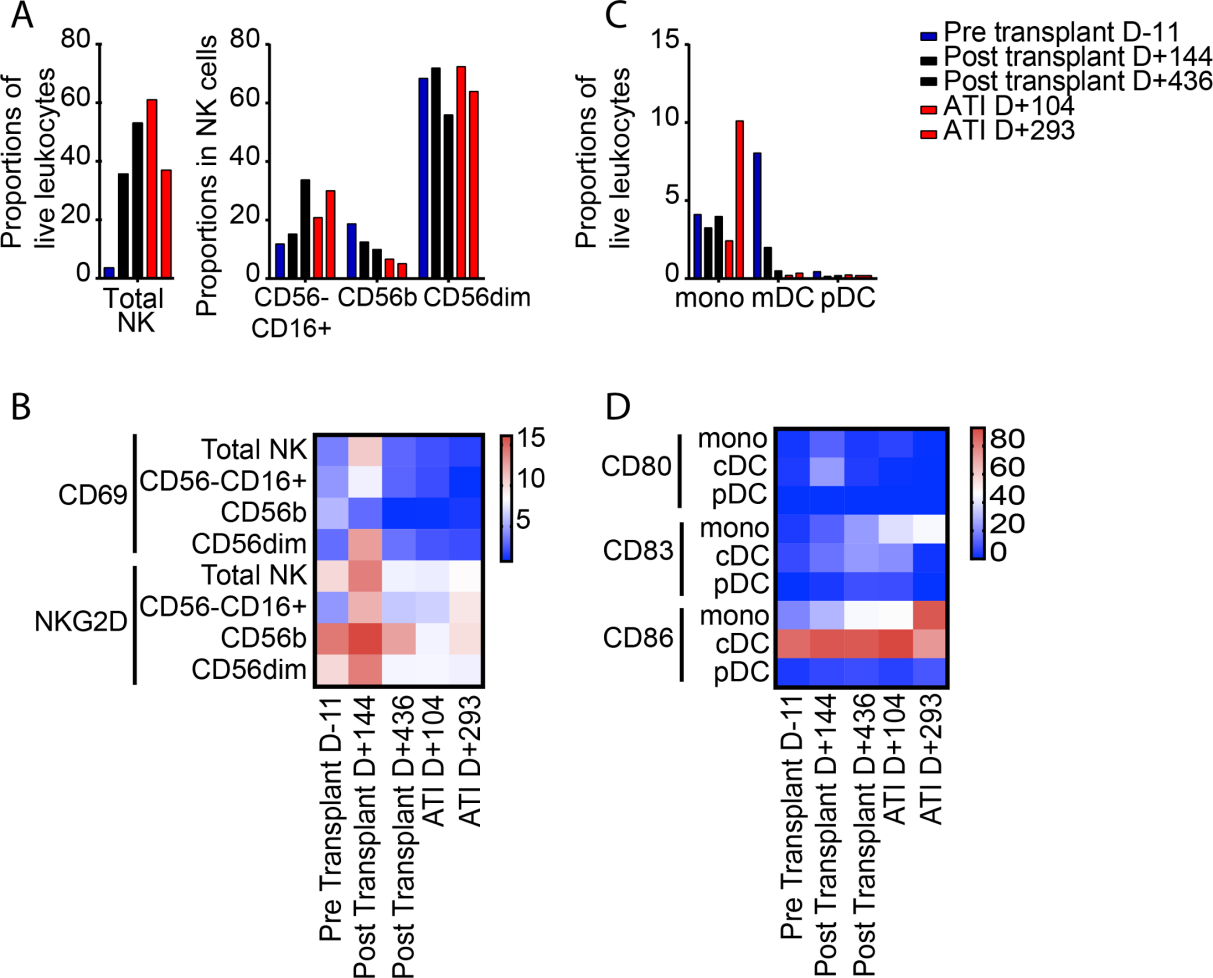
Longitudinal changes in innate immune cells. (A) Proportion of total NK cells and indicated NK cell subsets. Color coding reflects time of sample collection. (B) Spider diagram demonstrating longitudinal changes in proportions of NK cell subsets with indicated phenotypic characteristics. (C) Proportions of CD14+ monocytes, HLA-DR+ CD11c+ lin− myeloid dendritic cells, and HLA-DR− CD123+ plasmacytoid dendritic cells at indicated time points during treatment course. (D) Heatmap showing longitudinal evolution in the proportions of monocytes, conventional dendritic cells, and plasmacytoid dendritic cells expressing CD80, CD83, or CD86. ATI, analytic treatment interruption; cDC, conventional dendritic cells; mDC, myeloid dendritic cells; mono, monocytes; NK, natural killer; pDC, plasmacytoid dendritic cells.

Innate immune cells can modulate antiviral immune defense and HIV-1 immune activation by a variety of mechanisms [36]. To analyze change in the innate immune system during the patient’s treatment course, we focused on CD3− CD56+ natural killer (NK) cells, arguably the most important effector component of the innate immune system [37]. Before transplantation, the total number of NK cells was severely diminished, and consisted predominantly of CD56dim CD16− cells, while CD16+ CD56− NK cells, previously associated with improved cytotoxic function [37,38], made smaller contributions (Fig 6A). This relative distribution of NK cell subsets persisted during the subsequent disease course, although the total number of NK cells increased to normal levels. NK cell activation markers, in particular NKG2D, were again most strongly expressed at the time of GVHD on all NK cell subsets (Fig 6B); similar but less obvious trends were also noted for expression of NKp46 and NKp30 on NK cells (S1 Fig). Remarkably, CD11c+ myeloid dendritic cells were most frequently detected immediately prior to transplantation, and subsequently declined to levels more typically observed in ART-treated HIV-1-infected patients (Fig 6C); relative proportions of CD14+ monocytes and plasmacytoid dendritic cells remained relatively stable throughout the entire observation period, as did expression levels of co-stimulatory and dendritic cell maturation markers on these dendritic cells and monocytes (Fig 6D).

### B cell immune responses

**Fig 7:**
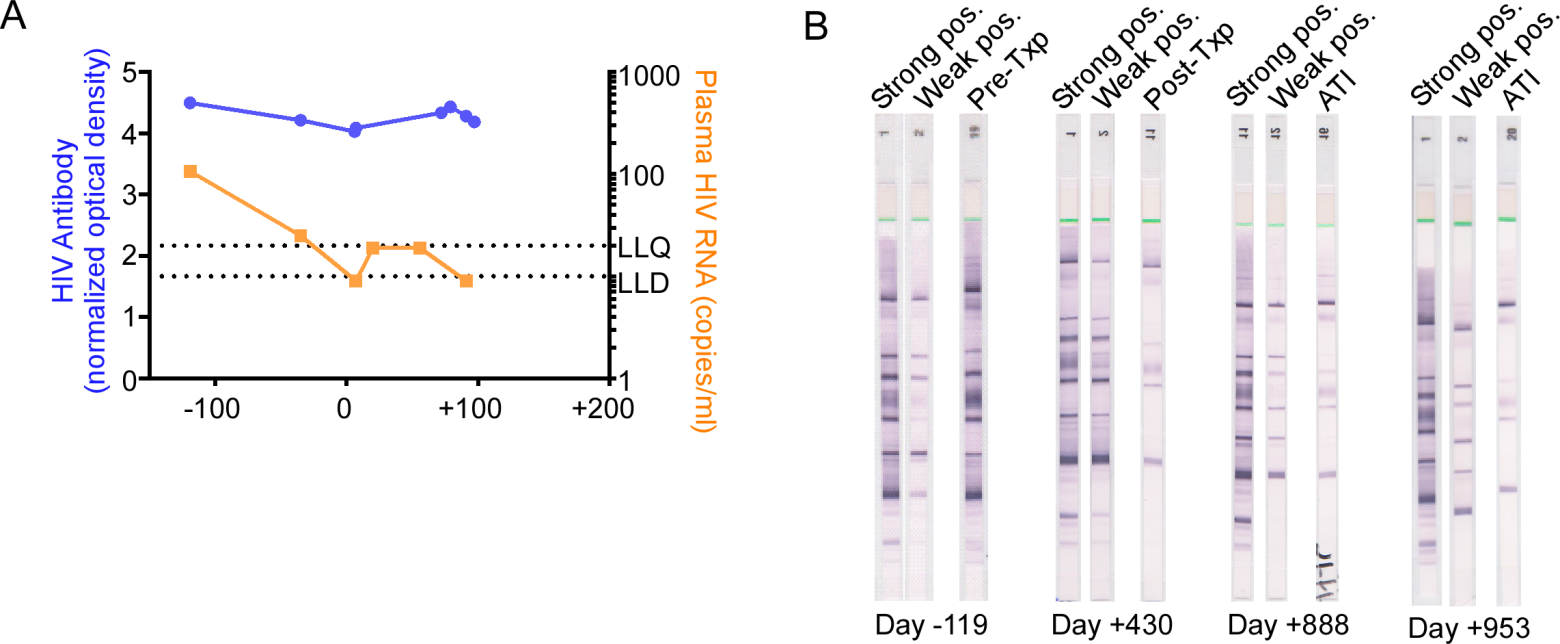
HIV-1 antibody assessment in the peri-transplant period. (A) HIV-1 antibodies in serum were quantified by the limiting antigen assay in the pre-transplant and early post-transplant period. (B) Anti-HIV-1 Western blot analyses were performed on the days indicated. “Strong pos.” and “Weak pos.” represent internal positive controls for the assay. ATI, analytic treatment interruption; Txp, transplant.

Proportions of total CD19+ B cells and non-switched memory B cells were smallest prior to transplantation, but levels normalized during the post-transplantation disease course (S2 Fig). Consistent with these data, but unlike the Berlin patient [5], quantitative levels of anti-HIV antibodies did not change significantly in the first 100 days after transplantation (Fig 7A). However, at later time points after transplant, our patient demonstrated declining levels of anti-HIV antibodies, as demonstrated by the decreasing number and intensity of anti-HIV bands on Western blot from day −119 to day +888 (Fig 7B).

## Discussion

To date, the only described cure of an adult with HIV-1 is the Berlin patient, who was cured of HIV-1 following treatment for acute myeloid leukemia that included induction chemotherapy and anti-thymoglobulin treatment, followed by 2 allo-SCTs from a donor with a homozygous *CCR5* Δ32 mutation [4]. Two Harvard patients who underwent reduced intensity conditioning and allo-SCT, and had significant reductions in the latent viral reservoir, eventually had virologic rebound off ART; these cases are cautionary examples that near eradication of the reservoir may not be sufficient to achieve even a functional cure [6]. We present extensive host and virologic studies on an additional HIV-1 positive individual who underwent allo-SCT. Table 3 compares clinical features between these 4 cases of prolonged ARV-free HIV-1 remission after allo-SCT. Results from our patient confirm that HIV-1 burden (as measured by total HIV-1 DNA and integrated HIV-1 DNA) can decline significantly after allo-SCT, but this is not necessarily accompanied by cure of HIV-1 infection.

**Table 3:** Clinical features of previous selected patients.

**Clinical feature**	**Mayo patient**	**Berlin patient**	**Harvard patient A**	**Harvard patient B**
Years of HIV-1 before Tx (years of ART)	23 years (9 cumulative years)	>10 years (4 years)	Lifelong (3–4 years)	20 years (7 years)
Viral load prior to Tx	23 copies/ml	Undetectable	Undetectable	Undetectable
Donor *CCR5* genotype	Wild-type	CCR5 Δ32 homozygous	Wild-type	Wild-type
Donor HLA	A*03,24; B*07,27; Cw*02,07	A*0201; B*0702,3501; Cw*0401,0702; DRB1*0101,1501; DQB1*0501,0602	A*0201,2301; B*4403,5101; Cw*0202,0401	A*02,24; B*08,1517; Cw*07,07
Recipient HLA	A*03,24; B*07,27; Cw*02,07	A*0201; B*0702,3501; Cw*0401,0702; DRB1*0101,1501; DQB1*0501,0602	A*0201,2301; B*4403,5101; Cw*0202,0418	A*02,24; B*08,1517; Cw*07,07
Conditioning regimen	Rituximab, cyclophosphamide, vincristine, doxorubicin, dexamethasone, methotrexate, cytarabine (4 cycles)	Cytarabine, gemtuzumab, rabbit anti-thymocyte globulin, whole-body radiation	Gemcitabine, navelbine, doxorubicin, busulfan, fludarabine	Busulfan, fludarabine
GVHD episodes (sites and grade)	Mouth and colon grade 1	Skin grade 1	Skin, eye, liver	Skin, liver, oropharynx
Duration of ART from Tx to ATI	3.2 years	0 (stopped 1 day before SCT)	4.3 years	2.6 years
Time from ATI to HIV-1 RNA rebound	288 days	>10 years	84 days	219 days
Hematologic malignancy	Acute lymphoblastic leukemia, B lineage, with myeloid features	Acute myeloid leukemia	Nodular sclerosing Hodgkin lymphoma	Diffuse large B cell lymphoma; mixed cellularity Hodgkin disease

ATI, analytic treatment interruption; GVHD, graft-versus-host disease; SCT, stem cell transplant; Tx, transplant.

The major barrier to HIV-1 cure is the latent viral reservoir, composed largely of resting memory CD4 T cells that carry stably integrated, replication-competent HIV-1 DNA [39,40]. The mechanisms by which HIV-1 persists are multifactorial (reviewed in [41]). After therapy with effective ARV medications, plasma HIV-1 RNA becomes undetectable; however, cellular and anatomic reservoirs of HIV-1 persist. The half-life of the resting HIV-1-infected CD4 T cell is estimated to be approximately 44 months [42], which predicts that over 60 years of ARV therapy would be required to eradicate the reservoir of HIV-1 in resting T cells, provided fully suppressive HIV therapy can be achieved and maintained. However, the existence of chronic or intermittent low-level viral replication replenishes the viral reservoir by infection of additional CD4 T cells [43], some of which become latently infected resting memory CD4 T cells. In addition, other mechanisms may exist to contribute to maintenance of latently infected T cells, including homeostatic proliferation and clonal expansion [13]. Therefore, therapies that target HIV-1 replication are alone insufficient to eradicate HIV-1, and other interventions that target and eradicate the latent viral reservoir will be needed to cure HIV-1.

It remains unknown what components of the Berlin patient’s treatment were responsible for his HIV-1 cure. Possibilities include 2 courses of myeloablative chemotherapy eradicating the HIV-1 reservoir, *CCR5* Δ32 donor cells being resistant to HIV-1 reinfection, and a “graft-versus-HIV” effect. Accordingly, a variety of approaches are being evaluated to recapitulate this only HIV-1 cure, including PBSCT, gene therapy to knockdown *CCR5* or other host factors required for HIV-1 replication, and chimeric antigen receptors expressed in autologous CD8 T cells designed to kill HIV-1-infected cells (reviewed in [44]).

In experimental models in which rhesus macaques infected with simian HIV underwent myeloablative conditioning and autologous stem cell transplantation, despite significant reductions in viral reservoir size, viral rebound occurred shortly after ART interruption [45]. A greater than 10,000-fold reduction of the HIV-1 reservoir in a host would be required to prevent HIV-1 rebound after discontinuing combination ART, according to stochastic modeling estimates [46], a goal that would be difficult to attain. The HIV-1 reservoir is also difficult to measure reliably, as currently available assays are insufficiently sensitive to detect such low levels of virus above background signals in the assays themselves. An upper limit estimate of the magnitude of HIV-1 reservoir reduction in our patient would be approximately 200-fold, which is consistent with a delay in viral rebound, assuming stochastic reactivation of a reduced number of latently infected cells [46,47].

Both the donor and the recipient involved in our case had wild-type *CCR*5. The donor for the Berlin patient was genotypically *CCR5* Δ32, which has inspired the exploration of gene therapy approaches for HIV-1 eradication, including knock-down of host proteins required for HIV-1 replication, such as CCR5, as well as studies to identify donors for hematopoietic stem cell transplantation who are homozygous for *CCR5* Δ32. In a pilot study, autologous CD4 T cells modified by CCR5 knock-down had a prolonged half-life compared to unmodified cells, demonstrating the feasibility of the approach, yet the functionality of the reinfused cells was not assessed, and approaches to replacing all potential target cells will need to be addressed for this approach to have an impact on effecting a cure [48].

A recent report of an HIV-1-infected patient who underwent allo-SCT from a homozygous *CCR5* Δ32 donor further confounds our understanding as that recipient experienced virologic rebound associated with a shift in viral tropism from CCR5-tropic to X4-tropic [49]. Thus, *CCR5* Δ32 transplants alone may not be sufficient for HIV-1 cure.

As the Berlin patient’s post-transplant course was complicated by GVHD, it has been hypothesized that allogeneic responses post-transplant may exert a graft-versus-HIV effect by killing residual recipient lymphocytes, including latently infected cells. In fact, our patient experienced grade I GVHD of the bowel that was managed symptomatically in an attempt to promote this effect. Coincident with clinical GVHD in our patient, immunologic studies of PBMCs at day +142 revealed increased expression of activation markers in multiple cell lineages, including NK cells, CD4 T cells, and CD8 T cells. Also coincident with clinical GVHD, cell-associated HIV-1 RNA was detectable at day +142, at levels comparable to pre-transplant levels (Fig 1B), despite total and integrated HIV-1 DNA being reduced and viremia being undetectable at the same time (Fig 2). Altogether, these data suggest that GVHD caused polyclonal and generalized immune activation and consequent viral production from latent sources. However, in the absence of demonstrable HIV-specific CD8 T cells (Fig 5A), and with waning B cell immunity to HIV-1 (Figs 7 and S2), GVHD may not have had an effective graft-versus-HIV effect, but instead may have promoted HIV-1 persistence by stimulating subclinical replication, potentially in tissue sanctuary sites, although this is speculative.

There are 3 main limitations to our study. First, we did not have access to archived samples from prior to presentation for leukemia evaluation. Therefore, we were unable to characterize long-term trends in HIV reservoir size and phylogeny before allo-SCT. Second, we primarily sampled blood cells, with only 1 lymphoid tissue sample analyzed. Therefore, we were unable to characterize the HIV reservoir contained in the lymphoid tissue. In addition, it is likely that the immunologic changes noted in Figs 5 and 6 were the result of the pre-transplant conditioning regimen, the transplant procedure, and/or GVHD, and not necessarily due to underlying HIV infection or clearance thereof. Finally, since this is a single case description, it is unclear if the findings are applicable to other HIV positive patients undergoing allo-SCT.

Despite these limitations, our case clearly illustrates that allo-SCT in the setting of ART-suppressed HIV-1 infection can significantly reduce the HIV-1 reservoir size, in this case to a level that was sufficiently low that viral rebound did not occur for 288 days following treatment interruption. It is noteworthy that once virus rebound did occur, the proviral sequence was phylogenetically different from the viral sequences identified in the peri-transplant period, and may have originated from sanctuary tissue sites harboring archived viral species seeded during the extensive HIV-1 disease process preceding the patient’s oncologic history. Most researchers in the HIV-1 cure field believe that successful cure of HIV will involve a combination of approaches that act by different mechanisms to synergistically eradicate viral reservoirs. Our data suggest that allo-SCT can profoundly reduce HIV-1 reservoir size, but incompletely, and raise the hypothesis that coupling allo-SCT with other viral reservoir reduction approaches might eventually enable a cure or long-standing remission of HIV-1 infection.

## Supporting information

S1 STROBE Checklist STROBE statement. (DOCX)

S1 Fig Detailed phenotypic characterization of NK cells, CD4 T cells, and CD8 T cells. Heatmaps for (A) NK cells, (B) CD4 T cells, and (C) CD8 T cells reflect proportions of cells with indicated phenotypic properties at given time points.
(DOCX)

S2 Fig Phenotypic characteristics of B cells in the described patient. (A) Longitudinal evolution of total B cells and indicated B cell subsets. Phenotypic classification was determined as follows: memory non-switched: CD27+ IgD+; memory IgM-only: CD27+ IgD− IgM+; memory switched: CD27+ IgD− IgM−; plasmablast: CD27high IgD− CD38high; transitional T1-T2: CD27− IgD+ CD10+ CD38high; memory double-negative: CD27− IgD−. (B) Heatmap reflecting the longitudinal proportion of B cells with indicated phenotypic characteristics.
(DOCX)

## Abbreviations

allo-SCT: allogeneic stem cell transplantation
ARV: antiretroviral
ATI: analytic treatment interruption
ddPCR: droplet digital PCR
GVHD: graft-versus-host disease
IUPM: infectious units per million cells
NK: natural killer
OD: optical density
PBMC: peripheral blood mononuclear cell
PBSCT: peripheral blood stem cell transplantation
QVOA: quantitative viral outgrowth assay
