# Supplementary material for: Extensive virologic and immunologic characterization in an HIV-infected individual following allogeneic stem cell transplant and analytic cessation of antiretroviral therapy: A case study
Source: PLoS Med. 2017 Nov 28;14(11):e1002461. doi: 10.1371/journal.pmed.1002461 (PMC5705162; doi:10.1371/journal.pmed.1002461)
Supplement: S1 STROBE Checklist — (DOCX) [file pmed.1002461.s001.docx]

STROBE Statement—Checklist of items that should be included in reports of ***cohort studies***

|  | Item No | Recommendation |
| --- | --- | --- |
| **Title and abstract** | 1 | (*a*) Indicate the study’s design with a commonly used term in the title or the abstract Title page, title |
|  |  | (*b*) Provide in the abstract an informative and balanced summary of what was done and what was found - Abstract page |
| Introduction | | |
| Background/rationale | 2 | Explain the scientific background and rationale for the investigation being reported Introduction, paragraphs 1-4 |
| Objectives | 3 | State specific objectives, including any prespecified hypotheses Introduction, paragraph 4 |
| Methods | | |
| Study design | 4 | Present key elements of study design early in the paper Methods, paragraph 1 |
| Setting | 5 | Describe the setting, locations, and relevant dates, including periods of recruitment, exposure, follow-up, and data collection Results, paragraphs 1-3 |
| Participants | 6 | (*a*) Give the eligibility criteria, and the sources and methods of selection of participants. Describe methods of follow-up Not applicable |
|  |  | (*b*) For matched studies, give matching criteria and number of exposed and unexposed Not applicable |
| Variables | 7 | Clearly define all outcomes, exposures, predictors, potential confounders, and effect modifiers. Give diagnostic criteria, if applicable Not applicable |
| Data sources/ measurement | 8* | For each variable of interest, give sources of data and details of methods of assessment (measurement). Describe comparability of assessment methods if there is more than one group Please see Methods section |
| Bias | 9 | Describe any efforts to address potential sources of bias Not applicable |
| Study size | 10 | Explain how the study size was arrived at Not applicable |
| Quantitative variables | 11 | Explain how quantitative variables were handled in the analyses. If applicable, describe which groupings were chosen and why Please see individual Methods sections for assays |
| Statistical methods | 12 | (*a*) Describe all statistical methods, including those used to control for confounding Not applicable |
|  |  | (*b*) Describe any methods used to examine subgroups and interactions Not applicable |
|  |  | (*c*) Explain how missing data were addressed Not applicable |
|  |  | (*d*) If applicable, explain how loss to follow-up was addressed Not applicable |
|  |  | (*e*) Describe any sensitivity analyses Not applicable |
| Results | | |
| Participants | 13* | (a) Report numbers of individuals at each stage of study—eg numbers potentially eligible, examined for eligibility, confirmed eligible, included in the study, completing follow-up, and analysed See Results, paragraph 1 |
|  |  | (b) Give reasons for non-participation at each stage Not applicable |
|  |  | (c) Consider use of a flow diagram Not applicable |
| Descriptive data | 14* | (a) Give characteristics of study participants (eg demographic, clinical, social) and information on exposures and potential confounders See Results, paragraph 1 and Table 1 |
|  |  | (b) Indicate number of participants with missing data for each variable of interest Not applicable |
|  |  | (c) Summarise follow-up time (eg, average and total amount) See Figure 1 |
| Outcome data | 15* | Report numbers of outcome events or summary measures over time See Figures 1-6 |
| Main results | 16 | (*a*) Give unadjusted estimates and, if applicable, confounder-adjusted estimates and their precision (eg, 95% confidence interval). Make clear which confounders were adjusted for and why they were included Not applicable |
|  |  | (*b*) Report category boundaries when continuous variables were categorized Not applicable |
|  |  | (*c*) If relevant, consider translating estimates of relative risk into absolute risk for a meaningful time period Not relevant |
| Other analyses | 17 | Report other analyses done—eg analyses of subgroups and interactions, and sensitivity analyses Not applicable |
| Discussion | | |
| Key results | 18 | Summarise key results with reference to study objectives Discussion, paragraph 1 |
| Limitations | 19 | Discuss limitations of the study, taking into account sources of potential bias or imprecision. Discuss both direction and magnitude of any potential bias Discussion, paragraph 8 |
| Interpretation | 20 | Give a cautious overall interpretation of results considering objectives, limitations, multiplicity of analyses, results from similar studies, and other relevant evidence Discussion, paragraph 9 |
| Generalisability | 21 | Discuss the generalisability (external validity) of the study results Discussion, paragraph 8 |
| Other information | | |
| Funding | 22 | Give the source of funding and the role of the funders for the present study and, if applicable, for the original study on which the present article is based Available with submission information |

*Give information separately for exposed and unexposed groups.

**Note:** An Explanation and Elaboration article discusses each checklist item and gives methodological background and published examples of transparent reporting. The STROBE checklist is best used in conjunction with this article (freely available on the Web sites of PLoS Medicine at http://www.plosmedicine.org/, Annals of Internal Medicine at http://www.annals.org/, and Epidemiology at http://www.epidem.com/). Information on the STROBE Initiative is available at http://www.strobe-statement.org.
