# Supplementary figures and images for: Extensive virologic and immunologic characterization in an HIV-infected individual following allogeneic stem cell transplant and analytic cessation of antiretroviral therapy: A case study
Source: PLoS Med. 2017 Nov 28;14(11):e1002461. doi: 10.1371/journal.pmed.1002461 (PMC5705162; doi:10.1371/journal.pmed.1002461)

**S1 Fig**

**
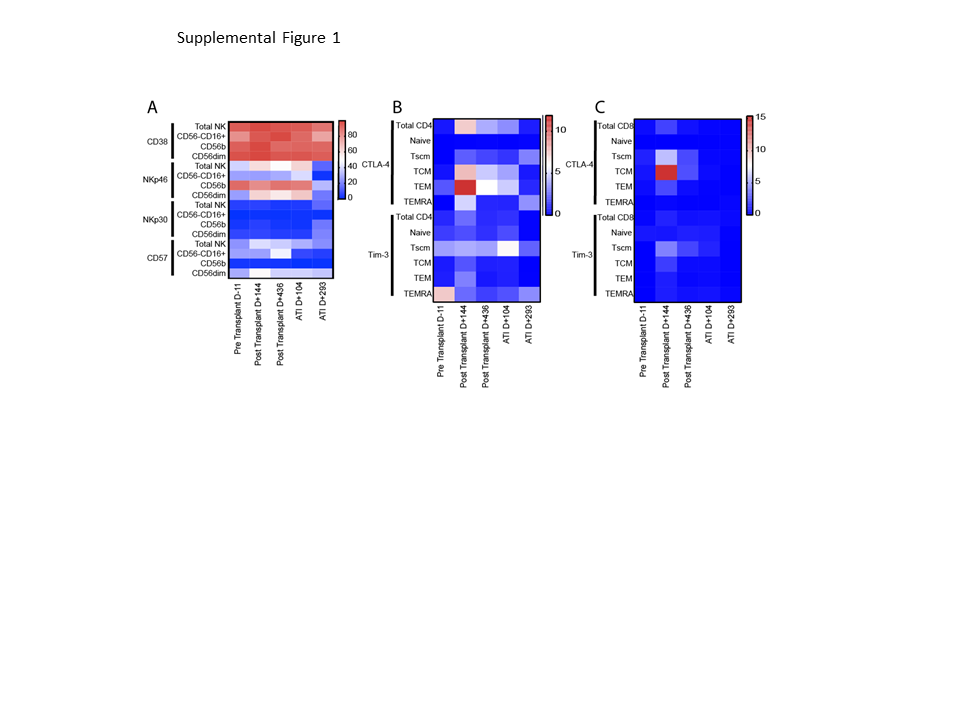
**

Supplement: S1 Fig — Heatmaps for (A) NK cells, (B) CD4 T cells, and (C) CD8 T cells reflect proportions of cells with indicated phenotypic properties at given time points. (DOCX) [file pmed.1002461.s002.docx]

**S2 Fig**

**
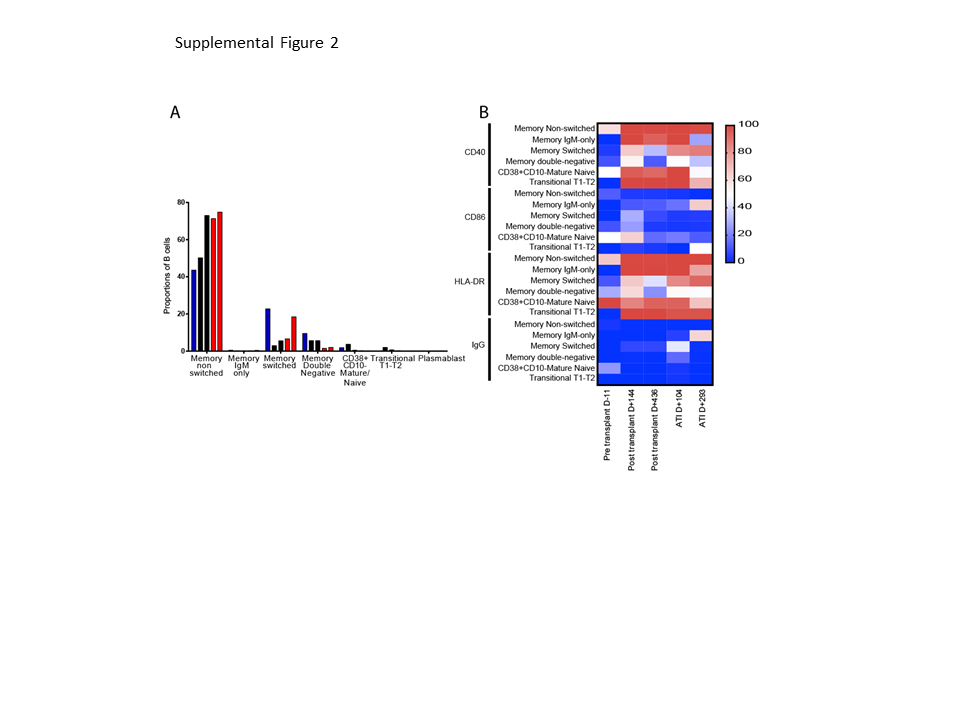
**

Supplement: S2 Fig — (A) Longitudinal evolution of total B cells and indicated B cell subsets. Phenotypic classification was determined as follows: memory non-switched: CD27+ IgD+; memory IgM-only: CD27+ IgD− IgM+; memory switched: CD27+ IgD− IgM−; plasmablast: CD27high IgD− CD38high; transitional T1-T2: CD27− IgD+ CD10+ CD38high; memory double-negative: CD27− IgD−. (B) Heatmap reflecting the longitudinal proportion of B cells with indicated phenotypic characteristics. (DOCX) [file pmed.1002461.s003.docx]
